# The Sterilization of Degenerate Criminals

**Published:** 1911-10

**Authors:** Tom A. Williams

**Affiliations:** M. D., C. M., Edin.; Washington, D. C.; Memb. Correp. Soc. de Nurologie, Paris; Memb. Correp. Soc. de Psychologie, Paris, etc.; Neurologist to Epiphany Dispensary


### THE STERILIZATION OF DEGENERATE CRIMINALS.

By Tom A. Williams, M. D., C. M., Edin., Washington, D. C.

*Memb. Correp. Soc. de Nurologie, Paris; Memb. Correp. Soc. de Psychologie, Paris, etc.; Neurologist to Epiphany Dispensary.*

Civilization has interfered with natural selection to an extent which has brought an acute realization of the danger of conditions under which the propagation of the unfit far exceeds that of those who are physically, intellectually, and morally of most advantage to the community. The parasites who prey upon society are a tremendous drain upon economic resources, which are becoming less and less sufficient to the needs of modern life. Utopians have long advocated the artificial elimination of the proved unfit; but they have always been met not only by the sentiment of humanitarianism, but with the shortcomings of present criteria for determining elimination. The blame for this rests largely upon the school of Lombroso, whose error that genius was a form of degeneracy has powerfully influenced sociologic thought.

More accurate inductions have, however, exploded a good many of Lombroso's hasty generalizations, of which this is not the least conspicuous. Even practical politicians have become aware of this; and in less conservative societies, psychiatrists have succeeded in inducing legislation for preventing the propagation of the hereditary criminal.

In the State of Indiana, 500 vasectomies of criminals have been performed since the compulsory law of 1907; and the success of the measures rests not only upon its social prophylactic value, but it has had an extraordinary effect upon the criminals themselves. It has been the rule that a morose, sullen, suspicious, erotic degenerate becomes after operation a sunny, bright, natural human being. Not only so, but Dr. Sharp of the Indiana penitentiary declares "that many boys have come to me and urged me to *fix them* as I had fixed some friend or associate of theirs." Masturbators have asked for castration, which I refuse, but substitute vasectomy, which cures many cases." Now similar laws have been passed in Utah, California, and Connecticut, and are being considered in New Jersey, Texas and Minnesota.

At the meeting of the Tri-State Medical Association at Richmond in February, a unanimous resolution was sent to the Virginia legislature then in session to endorse a bill then before it, making it compulsory for each Institution which cares for criminals, idiots and imbeciles to add to their staff a skilled surgeon, an alienist and the Secretary of the state board of charity and direction, who shall examine with a view to sterilization those inmates recommended by the physicians and managers of such an Institution.

Dr. Carrington, who is largely responsible for the measure, believes that eventually the precedure will be legally extended to perverts, the feeble-minded and other defectives, even although not criminals; for his observations are that when the patients observe the absence of ill-effects and indeed an improvement of health, they are content to forego procreation.

Although of negative aspects as regards eugenics, these phophylaxes cannot but accustom people's minds to the necessary positive point of view that for the improvement of the human race selection is required as well as elimination, and that the economic and social artificialities which interfere wth this must be abolished wherever a nation desires to survive in what has now become an intense strugle for existence.

The New England family of Edwards is a striking illustration. Descended from strong, religious ancestors, 1884 were traced through five generations. Of these 295 were college graduates; 13 college Presidents, 65 professors 60 doctors, 100 missionaries and clergymen, 175 officers of navy, 60 prominent authors, over 100 noted lawyers, 31 judges, 80 held public offices, 3 United States Senators, 15 railway presidents, several State Governors, members of Congress, Mayors of Cities, Ambassadors, Vice President, and *not one single convict.* What a contrast with the well-known Jukes family.

1758 K. St.

